# Escalating Oral Treprostinil Dose With Intravenous Treprostinil Bridging Therapy

**DOI:** 10.7759/cureus.54184

**Published:** 2024-02-14

**Authors:** Vanessa C Perez, Veronica Williams, Franck F Rahaghi

**Affiliations:** 1 Department of Pharmacy, Cleveland Clinic Florida, Weston, USA; 2 Department of Pulmonary and Critical Care Medicine, Cleveland Clinic Florida, Weston, USA

**Keywords:** right-sided heart failure, pulmonary vascular disease, clinical pharmacokinetics, primary pulmonary hypertension, pulmonary arterial hypertension, treprostinil

## Abstract

Oral treprostinil, approved for the treatment of pulmonary arterial hypertension, remains an attractive option in combination with other medications to delay disease progression and improve exercise capacity. However, patients are often challenged with the ability to overcome adverse effects as outpatients and reach effective doses in a timely manner. We describe a case of a 47-year-old female on oral treprostinil who presented to the clinic with worsening symptoms of disease, necessitating higher dosing. This patient was previously uptitrated outpatient with oral treprostinil, which had allowed her to remain stable for years. Once uptitrated with additional intravenous therapy, the oral treprostinil dose was gradually further increased to the new goal dosage, resulting in improvements in symptoms and right ventricular function. This case highlights the versatility of dose optimization of oral treprostinil with rapid bridging through intravenous therapy.

## Introduction

Transitioning from parenteral treprostinil to oral treprostinil in the inpatient setting, while not entirely novel as a clinical endeavor, remains a nebulous territory. Limited data are available to guide clinicians to a standardized protocol while adjusting for unique patient scenarios and tolerance for prostanoid therapy, with success noted in an earlier case detailed by Gleason et al. [1]. Similarly, patients established as outpatients on oral treprostinil therapy may experience challenges achieving their optimal dose, including adverse effect profile and potentially protracted time course to goal dosage. Patients may be limited by side effects when increasing dosage, which may warrant re-challenging of additional parenteral therapy in a supervised hospital setting. We present a unique case of a patient on oral treprostinil who was admitted for the addition of intravenous treprostinil in an inpatient setting with a rapid successful transition to increased oral therapy.

## Case presentation

The patient was a 47-year-old female with a past medical history of pulmonary hypertension, systemic lupus erythematosus, and chronic kidney disease stage 3. She was diagnosed in 2006 with WHO group 1 pulmonary hypertension and initiated on sildenafil, which she did not tolerate and was subsequently switched to bosentan and iloprost. In 2010, she received off-label stem cell transplantation abroad with partial transient relief for months and was restarted on sildenafil upon her return. Due to insurance limitations, the patient was unable to afford her medications and, in late 2011, became a candidate for the AMBITION trial [2]. Upon completion of the trial in 2014, the patient and physician were unblinded and switched to the trial medication consisting of combination therapy of ambrisentan 10 mg daily and tadalafil 20 mg every eight hours with relative clinical stability for some time. In 2018, she presented to our clinic feeling slightly fatigued with weight gain and occasional hypotension. The echocardiogram showed moderate right ventricular (RV) dilation but preserved systolic function and estimated right ventricular systolic pressure (RVSP) of 66 mmHg. Her six-minute walk test was in excess of 490 meters. She was initiated on riociguat 2.5 mg daily, discontinued off of tadalafil, and switched from ambrisentan to macitentan based on insurance formulary. She remained on macitentan and riociguat up until September 2020, when she was seen in the clinic, and the recommendation was made to add oral treprostinil to her regimen. She began therapy with titration of her dose every two weeks with a goal dose of 5 mg three times daily.

However, once at goal, she began to experience diarrhea, vomiting, jaw pain, and headaches and was subsequently decreased to 4.75 mg three times daily. Despite triple therapy, right heart catheterization demonstrated pulmonary vascular resistance of 8.4 Woods units (WU) with a normal wedge and preserved cardiac output. The subsequent echocardiogram two months later revealed severe RV dilation with moderately reduced RV systolic function; RVSP was noted to be 83 mmHg with right atrial pressure of 3 mmHg (Video 1). Tricuspid annular plane systolic excursion (TAPSE) was borderline at 20 mm. The N-terminal pro-B-type natriuretic peptide (NT-proBNP) was elevated to 304 pg/ml. Based on these findings and ongoing symptoms of exertional dyspnea and dizziness, our patient was directly admitted to the hospital to initiate intravenous (IV) treprostinil and further subsequent conversion to a higher oral dose.

**Video 1:** Initial echocardiogram demonstrating severe RV dilation and mild RV dysfunction prior to treprostinil titration. RV: right ventricular.

**Figure 1:**
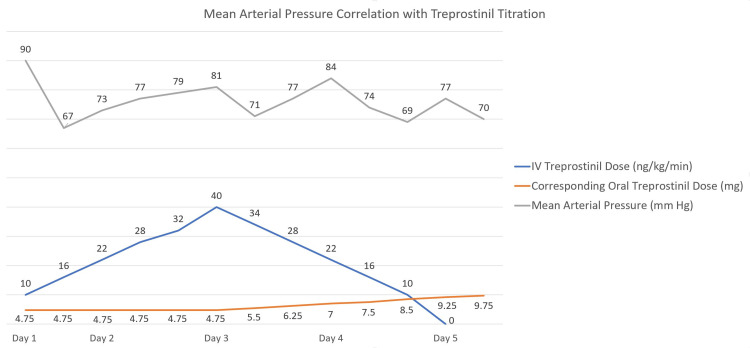
Mean arterial pressure correlation with treprostinil titration.

She was started on IV treprostinil along with her other pulmonary hypertension medications, which consisted of oral treprostinil 4.75 mg every eight hours, macitentan 10 mg daily, and riociguat 2.5 mg daily. IV treprostinil was started at 4 ng/kg/min and increased by 4 ng/kg/min every eight hours. Within just under 59 hours, she was titrated up to 40 ng/kg/min on the IV treprostinil. She remained hemodynamically stable with minimal side effects at this dose; thus, we began to increase her oral treprostinil. We reduced the IV treprostinil dose by 6 ng/kg/min every eight hours while concomitantly increasing her oral dose by 0.75 mg until she reached her new oral goal of 9.75 mg every eight hours (Figure 1).

Overall, the patient tolerated the transition well and side effects were treated as necessary, reported as intermittent headaches mediated by acetaminophen-caffeine-butalbital, nausea treated with ondansetron, and occasional anxiety treated with lorazepam.

She was evaluated post-discharge in the clinic with reports of mild jaw pain, nausea, and headache, which were managed supportively. Repeat echocardiogram showed recovered and normalized RV function with decreased RVSP to 67 mmHg compared to 83 mmHg prior (Video 2). Her NT-proBNP improved to 240 pg/ml and her six-minute walk test additionally improved to 640 meters.

**Video 2:** Four-chamber apical view demonstrating right ventricular dilation but normal right ventricular function improved from the prior study prior to the escalation of therapy.

## Discussion

Treprostinil first received approval for intravenous or subcutaneous use from the FDA in May of 2002. Later, in 2013, the FDA approved the oral extended-release tablets of treprostinil as an alternative option for patients with pulmonary arterial hypertension [3]. While some side effects are similar between the various routes, including headache, diarrhea, nausea, jaw pain, and flushing, the oral route offers obvious advantages [4]. Parenteral infusions have been associated with bloodstream infections and sepsis from indwelling central catheter use, thrombophlebitis, and site pain reactions. Additionally, the parenteral prostacyclin delivery system can pose a significant burden on the quality of life for these patients.

Oral prostacyclin therapy, while clearly convenient and physiologically beneficial, also poses unique challenges to patients and providers [5]. The safety profile demonstrated in the FREEDOM-EV trial is consistent with previous studies, which estimated adverse event rates at least 5% higher on oral treprostinil therapy compared to placebo [6]. Of the most common side effects, headache occurred in 75% of patients, diarrhea in 69%, flushing in 45%, nausea in 40%, and vomiting in 36%. In this study, 19% of patients on oral treprostinil therapy were discontinued from treatment due to adverse effects. Several strategies are reported in the literature to help patients mitigate adverse events to achieve higher doses. Some of the proposed strategies include reducing dosages and slowing titration. However, this can prove burdensome in patients who present with worsening symptoms when the time to higher dosing is critical. This was perhaps most evident in those patients who were previously receiving twice daily dosing of oral treprostinil in the study by Chakinala et al., wherein the dosing was changed to three times daily as part of an amended protocol to mitigate peak and trough drug differences with improved tolerability [7]. Another study by White et al. further demonstrated that a three times daily regimen for oral treprostinil provides reduced peak-to-trough ratios, which in turn improves tolerability to achieve goal doses [8]. Nonetheless, predicting a patient’s tolerability of oral treprostinil, in comparison to IV formulations, remains difficult, with speculation of a pharmacokinetic relationship to adverse effect incidence. We present a unique strategy to overcome the barriers encountered by adverse effects to achieve optimal dose despite using a three times daily regimen strategy.

As aforementioned, Chakinala et al. have described a successful total transition from parenteral to oral treprostinil therapy utilizing a standardized protocol over five days for most patients. Rapid titration of IV treprostinil in a hospital setting followed by conversion to oral treprostinil was previously described by our group in 2015 [1] in a case of a patient unable to be placed on IV or subcutaneous treprostinil but in need of high-dose prostacyclin therapy. The EXPEDITE study [9] explored time-to-goal dosing through a prospective, multicenter, open-label study in evaluating whether a short-term course of IV treprostinil can assist patients in quickly achieving optimal goal dose of treprostinil, through various strategies, including rapid inpatient, moderately rapid outpatient, and gradual outpatient. The data from these studies are promising in terms of expediency and safety of rapid titration and attainment of goal dosing as opposed to de novo initiation of oral treprostinil therapy. Here, we presented the case of a patient who was already on oral treprostinil, and inpatient rapid up-titration and bridging to additional oral treprostinil allowed for the achievement of the required dose, resulting in improvement of symptoms and RV function.

## Conclusions

The management of an already complex disease state such as pulmonary hypertension can be further complicated by the challenges of medication side effects and dose optimization. Our patient case describes a novel role for intravenous treprostinil used as a bridge to achieve therapeutic doses of oral treprostinil on a patient already established on oral therapy and should be considered as a viable therapeutic option for patients in similar circumstances.

## References

[1] Gleason JB, Dolan J, Piran P, Rahaghi FF (2015). The rapid initiation, titration, and transition from intravenous to oral treprostinil in a patient with severe pulmonary arterial hypertension. Case Rep Pulmonol.

[2] Galiè N, Barberà JA, Frost AE (2015). Initial use of ambrisentan plus tadalafil in pulmonary arterial hypertension. N Engl J Med.

[3] (2019). Orenitram. Research Triangle Park, NC: United Therapeutics Corporation.

[4] Kumar P, Thudium E, Laliberte K, Zaccardelli D, Nelsen A (2016). A comprehensive review of treprostinil pharmacokinetics via four routes of administration. Clin Pharmacokinet.

[5] Khan A, White RJ, Meyer G (2022). Oral treprostinil improves pulmonary vascular compliance in pulmonary arterial hypertension. Respir Med.

[6] White RJ, Jerjes-Sanchez C, Bohns Meyer GM (2020). Combination therapy with oral treprostinil for pulmonary arterial hypertension. A double-blind placebo-controlled clinical trial. Am J Respir Crit Care Med.

[7] Chakinala MM, Feldman JP, Rischard F (2017). Transition from parenteral to oral treprostinil in pulmonary arterial hypertension. J Heart Lung Transplant.

[8] White RJ, Parikh K, Allen R (2019). EXPRESS: long term study of oral treprostinil to treat pulmonary arterial hypertension: dosing, tolerability, and pharmacokinetics. Pulm Circ.

[9] Kingrey JF, Miller CE, Franco V (2023). Implementing the EXPEDITE parenteral induction protocol: rapid parenteral treprostinil titration and transition to oral treprostinil. Pulm Circ.

